# Contributions to Pathology and Therapeutics, with Especial Reference to Surgery

**Published:** 1843-07

**Authors:** 


Art. III. — *Beiträge zur Pathòlogie und Therapie mit besonderer Berucksichtigung der Chirurgie von* Dr. Carl Emmert, Privat docenten in Bern. Erstes Heft. 1842. 8vo, pp. 184.

*Contributions to Pathology and Therapeutics, with especial reference to Surgery.* By Charles Emmert, m.d. Private Teacher at Bern. Part I. 1842.

This is the first part of a series which it is the intention of the writer to publish annually, consisting of essays on the present condition of medicine and surgery, as well as on some of the principles of pathology, with accounts of and observations on individual cases of disease.

The present number contains a short account of the present condition of the healing art in Germany, with an essay on inflammation and hyperemia. To the former a small space only is devoted, whilst to the latter the author appears to have paid considerable attention. This part contains a summary of the views, especially microscopical, of the various observers of this subject with the addition of new experiments, and a laborious summary by the writer himself. The remaining part of the work is devoted to the consideration of individual cases, of which the following is a brief summary :

1. The removal of a large bursa from the external surface of the patella with complete success.

2. Division of the ligament on the under surface of the joint between the first and second phalanx of the second toe, as well as the flexor tendons with complete relief to contraction of that joint.

3. Removal of the fleshy growths for the cure of an inverted nail. This paper is also very valuable for the numerous references to the labours of others.

4. Recurrence of a medullary tumour on the arm after its third removal with pulmonary disease.

5. Aneurismal dilatation of the aorta immediately above the valves, with the formation of a second sac between the pericardium and aorta, and a third sac under the skin ; the first and second sac communicating by a round opening, the second and third sacs communicating by an opening between the 3d, 4th, and 5th ribs.

6. The most interesting case in the whole work is the description of a case of effusion of blood to the amount of two to three pounds in the thigh after a fall. The sac was laid open, and the wound examined without finding the bleeding vessel, subsequent to which the parts healed and the patient recovered.

